# The association of choroidal structure and its response to anti-VEGF treatment with the short-time outcome in pachychoroid neovasculopathy

**DOI:** 10.1371/journal.pone.0212055

**Published:** 2019-02-14

**Authors:** Keiko Azuma, Xue Tan, Shotaro Asano, Kimiko Shimizu, Asako Ogawa, Tatsuya Inoue, Hiroshi Murata, Ryo Asaoka, Ryo Obata

**Affiliations:** 1 Department of Ophthalmology, Graduate School of Medicine and Faculty of Medicine, The University of Tokyo, Tokyo, Japan; 2 Department of Ophthalmology, Tokyo Shinjuku Medical Center, Tokyo, Japan; Purdue University, UNITED STATES

## Abstract

Pachychoroid neovasculopathy (PNV) shares some anatomical features with other pachychoroid spectrum diseases, but little is known about the characteristics on the treatment with anti-vascular endothelial growth factor (VEGF). We investigated the effect of choroidal structure and responses to anti-VEGF on the prognosis of pachychoroid neovasculopathy (PNV) and other types of neovascular age-related macular degeneration (non-PNV). Twenty-one eyes with PNV and 34 eyes with non-PNV who had anti-VEGF treatment were retrospectively reviewed. Choroidal neovascularization (CNV) area at baseline was measured with fluorescein angiography (FAG). The luminal and stromal area in the choroid was measured by enhanced-depth-imaging (EDI) OCT at baseline and 1 month. The association between dry macula or LogMAR VA (visual acuity, VA) at 1 month and baseline values or changes in the luminal or stromal area at 1 month, baseline CNV area, or anti-VEGF drugs were analyzed in patients with or without PNV. In non-PNV, change of luminal area (coefficient = 7.0×10^−5^, p = 0.0001), baseline CNV area (coefficient = 0.18, p = 0.033), and aflibercept vs. ranibizumab (coefficient = 0.29, p = 0.0048) were chosen as predictors for dry macula by the model selection. Similarly, in non-PNV, change of luminal area (coefficient = 6.1×10^−6^, p = 0.033), baseline CNV area (coefficient = 0.034, p = 0.022), and aflibercept vs. ranibizumab (coefficient = 0.056, p = 0.0020) were chosen as predictors for greater VA improvement. In PNV, however, none of these factors was chosen as predictors for dry macula or VA improvement by the model selection. The result of the present study implied that structural response after anti-VEGF might be different between non-PNV and PNV in the treatment with anti-VEGF agents.

## Introduction

Age-related macular degeneration (AMD) is a major cause of progressive visual impairment in the developed world [1, 2]. Especially, choroidal neovascularization (CNV) in neovascular AMD (nAMD) is responsible for most AMD-related severe vision losses.

Recent studies have revealed that there is a subtype of nAMD with distinct characteristics, described as pachychoroid neovasculopathy (PNV) [3, 4]. PNV shares some anatomical features with other pachychoroid spectrum diseases, such as central serous chorioretinopathy (CSC) or pachychoroid pigment epitheliopathy (PPE), including diffuse or focal thickening of the choroid, absence of or only very few drusen, choroidal vascular hyperpermeability, retinal pigment epithelium (RPE) abnormalities irrespective of CNV, and dilated choroidal vessels [3, 5], suggesting that aberrant choroidal circulation may play an essential role in the pathogenesis of PNV [3, 4].

Previous studies have shown different clinical findings between PNV and non-PNV. Compared to patients with nAMD without PNV (non-PNV), PNV occurs in younger patients [6]. Polypoidal lesion is similarly observed in PNV eyes (56%) compared to in non-PNV eyes (42%) [6, 7]. However, PNV patients had low genetic susceptibility to AMD, implicating the genetic background of the etiology of PNV might be different from that of non-PNV [6].

The response of PNV [or eyes with choroidal vascular hyperpermeability (CVH)] to anti-vascular endothelial growth factor (anti-VEGF) treatment is thought to be different from that of non-PNV. Patients with PNV are more likely to show persistent fluid after three loading doses of ranibizumab than non-PNV patients [8]. In contrast, in another study, PNV showed a longer pretreatment-free period than non-PNV [6]. In terms of visual outcome, patients with PNV treated by anti-VEGF drugs showed poorer visual improvement than non-PNV patients [9, 10]. A distinct response to anti-VEGF treatment in PNV implied that the relationship between disease activity of PNV and VEGF might be different from that of non-PNV. Of note, intraocular VEGF concentrations in PNV were not increased compared to those of non-PNV [11, 12]. The VEGF concentration at baseline predicted short-term treatment effects in non-PNV, but not in PNV [11]. PNV and non-PNV differed in pathogenesis and therapeutic effects, and in particular, differences in the structure of the choroid was distinguished between PNV and non-PNV. Therefore, we examined the relationship between their prognoses and choroid structures.

To the best of our knowledge, this is the first report to study anatomical changes of the choroidal structure and its association with dry macula or visual function in PNV or non-PNV eyes treated with anti-VEGF agents.

The purposes of the present study were (1) to assess the short-term response of choroidal structure to anti-VEGF treatment using enhance-depth imaging (EDI) optical coherence tomography (OCT) and (2) to characterize the differences between PNV and non-PNV.

## Methods

**Fig 1:**
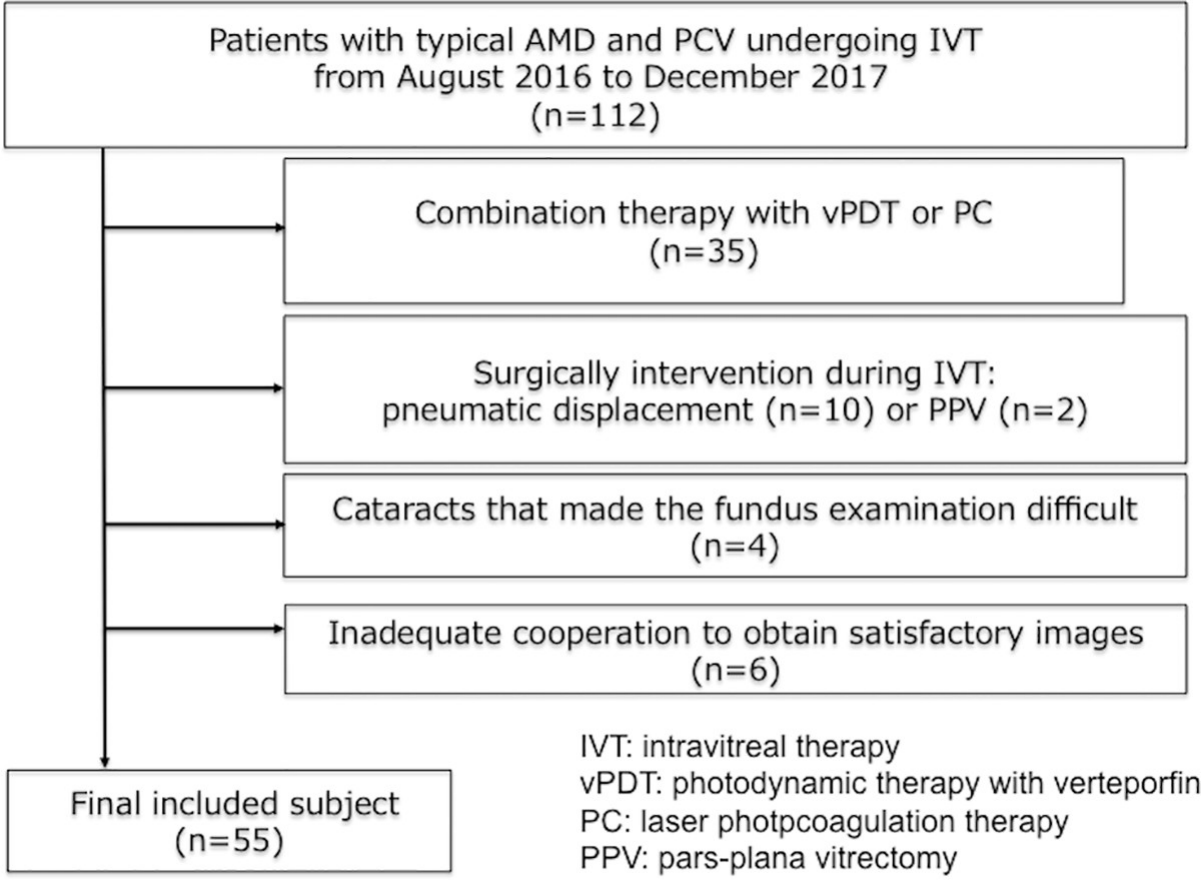
Flow charts of patients who met exclusion criteria for the present study. The selection was carried out using 112 AMD and PCV patients undergoing ranibizumab or aflibercept between the period of August 2016 and December 2017, at the outpatient clinic of Tokyo University Hospital.

This was a retrospective study that was approved by the Institutional Review Board (IRB) of the University of Tokyo. All data were fully anonymized before we accessed them. Written informed consent was not required by the IRB but participants who did not grant authorization to use their medical records for the research were excluded from analyses. We retrospectively reviewed the medical history of patients with typical AMD and PCV undergoing intravitreal injections of anti-VEGF from August 2016 to December 2017 at the outpatient clinic of Tokyo University Hospital. All patients underwent single intravitreal injection of an anti-VEGF drug, ranibizumab or aflibercept and were examined at their visit after one month. They underwent the ancillary multimodal imaging, i.e., OCT (Heidelberg Engineering GmbH, Dossenheim, Germany) using the EDI technique. Inclusion criteria were patients > 50 years of age with treatment and treatment-naïve CNV confirmed by fluorescein dye leakage on angiogram and the presence of one of the following on OCT: subretinal fluid, intraretinal fluid, or sub-RPE fluid. Patients who had already been treated with anti-VEGF before the data was acquired for analysis were considered as “having a treatment history”. The exclusion criteria were diagnosis of other pathologies (i.e., chorioretinal atrophy in the macula, organic eye diseases, previous laser treatment, cataracts that made the fundus examination difficult, glaucoma, and any other retinal disorders) and history of other treatments than intravitreal anti-VEGF drug administration such as photodynamic therapy, laser photocoagulation for AMD. Also, the exclusion criteria included inadequate cooperation to obtain satisfactory images. The flowcharts of patient selection were shown in **Fig 1**.

Areas of CNV were measured with fluorescein angiography before intravitreal anti-VEGF injection. The choroidal area on the fovea was assessed using EDI-OCT (HRA Spectralis; Heidelberg Engineering, Dossenheim, Germany). Binarization was performed separately using the methods of Sonoda et al [13], and we followed detailed algorithms for their technique. Binarization of subfoveal choroidal areas at the width of 1,000 μm centered on the fovea was performed by a modified Niblack method using Image J software. The definition of the ROI and the analysis were independently made by two masked graders. Then, the images were converted to 8 bits, and Niblack auto-local threshold was applied to binarize the images to separate luminal and stromal choroidal areas. The selection and analyses were made by two masked graders, independently (KA and SA). The intra-grader agreement was high, with an intraclass correlation coefficient of 0.74 [95% confidence interval (CI) 0.56–0.85] for the CNV area before treatment. The intra-grader agreement was very high, with an intraclass correlation coefficient of 0.70 (95% CI 0.50–0.83) for the luminal area before treatment, and 0.90 (95% CI 0.81–0.94) after treatment [14].

### Definition of PNV

In this study, PNV was diagnosed if all of the following criteria were satisfied: (1) CNV in either eye; (2) subfoveal choroidal thickness ≥ 200 μm in both eyes; (3) no drusen or only non-extensive (total area, ≤ 125 μm circle) hard drusen (≤ 63 μm) in both eyes (AREDS category 1, no AMD); (4) CSC or PPE characteristics; namely, choroidal vascular hyperpermeability, retinal pigment epithelium (RPE) abnormality independent of CNV lesions, the presence of dilated choroidal vessels or thickening below the Type 1 CNV, or a history of CSC [6, 15]. Other eyes were classified as non-PNV.

### Statistical analysis

The baseline characteristics or their change before and after the treatment in patients with PNV and non-PNV was analyzed using the chi-squared test for categorical variables and *t*-test for continuous variables. The proportion of patients with “Dry macula,” which was defined as absence of intraretinal or subretinal fluid on OCT and absence of hemorrhage by funduscopy based on the previous report [16] was compared between PNV and non-PNV using chi-square test. The visual change one year after the treatment was also reviewed. The relationship between the LogMAR visual acuity (VA) change at 1 month, and the nine explanatory variables (age, sex, treatment history, anti-VEGF: ranibizumab or aflibercept treatment, the area of CNV before treatment, choroidal structure: baseline value or change of luminal and stromal area) were evaluated using a linear model in PNV or non-PNV. The optimal linear model was then selected among all possible combinations of predictors: 2^9^ patterns, based on the second order bias corrected Akaike Information Criterion (AICc) index (denotes optimal model). We used correlation and multiple regression cross-sectional analyses to determine associations of baseline parameters or changes in 1-month parameters.

The AIC is a well-known statistical measure used in model selection, and the AICc is a corrected version of the AIC, which provides an accurate estimation when the sample size is finite [17]. The degrees of freedom in a multivariate regression model decreases with a large number of variables. It is therefore recommended that clinicians use the model selection method to improve the model fit by removing redundant variables [18, 19]. These processes were iterated only in the PNV and non-PNV patients.

All statistical analyses were carried out using the statistical programing language “R” (version 3.1.3; The R Foundation for Statistical Computing, Vienna, Austria).

## Results

We retrospectively reviewed 55 eyes of 55 patients with PNV and non-PNV. There were 21 eyes with PNV and 34 eyes with non-PNV. Among 55 patients in total, 89% of cases had a history of anti-VEGF treatment, and 11% was treatment-naïve patients. The baseline characteristics of the patients before treatment are shown in **Table 1**.

**Table 1:** The baseline characteristics of 55 Japanese patients with PNV or non-PNV.

Variables	Total	PNV	non-PNV	P value
**Number of eyes**	55	21	34	
**Age (mean ± SD, years)**	75.8 ± 7.7	73.7 ± 9.8	77.3 ± 6.5	0.12
**Sex/ Male (%)**	39 (71%)	15 (71%)	24 (71%)	0.94
**Treatment history (yes)**	49 (89%)	18 (86%)	31 (91%)	0.54
**Typical AMD (%)**	65	48	76	0.03
**PCV (%)**	35	52	24	
**LogMAR VA (mean ± SD)**	0.41 ± 0.39	0.54 ± 0.39	0.33 ± 0.37	0.06
**CNV (mm, mean ± SD)**	1.40 ± 0.91	1.43 ± 1.05	1.39 ± 0.83	0.86
**Luminal area** **(**μ**m** ^**2**^**)**	57780.2	69413.1	50595.2	0.0011
**Stromal area (**μ**m** ^**2**^**)**	36039.0	42093.7	32299.4	<0.001
**CCT (μm, mean ± SD)**	236 ± 96	306 ± 80	193 ± 79	<0.001

The average luminal area was 69,413 μm^2^ (range, 21,575–122,367 μm^2^) in the PNV group and 50,595 μm^2^ (range, 19,856–96,899 μm^2^) in the non-PNV group. Patients with PNV had significantly larger luminal areas than those with non-PNV (p = 0.0011, Wilcoxon signed-rank test). The average central choroidal thickness (CCT) was 306 μm (range, 201–507μm) in the PNV group and 193 μm (range, 56–392 μm) in the non-PNV patients. The CCT in PNV was significantly larger than those in non-PNV. At baseline, all patients showed intraretinal/subretinal fluid or hemorrhage. Their visual and anatomical outcomes are shown in **Table 2**.

**Table 2:** Visual and anatomical outcomes in patients with PNV (N = 21) or non-PNV (N = 34) before and after treatment.

Variables	Total	PNV	non-PNV	P value
**Baseline LogMAR VA**	0.41 ± 0.39	0.54 ± 0.39	0.33 ± 0.37	0.06
**LogMAR after 1 month**	0.38 ± 0.39	0.48 ± 0.42	0.32 ± 0.30	0.13
**LogMAR change (range)**	-0.03 (-0.056 to 0.0017)	-0.05 (-0.12 to 0.011)	-0.011 (-0.04 to 0.016)	0.17
**Dry macula (%)**	67	81	59	0.092
**Luminal area change (%)**	-3	-4	-1	0.093
**Stromal area change (%)**	1.5	2	1	0.60

The best-corrected visual acuity (BCVA) improvements at 1 month did not significantly differ between the two groups. Dry macula rate was 67% in total, 81% in PNV, and 59% in non-PNV. There was no significant difference between the PNV and non-PNV patients in dry macula rate. The visual change in PNV and non-PNV after one year were -0.07±0.22 and -0.07±0.27, respectively. There was no difference between the two groups.

As shown in **Table 3**, in the optical model for dry macula in non-PNV, anti-VEGF (aflibercept, coefficient = 0.19, p = 0.047), CNV area at baseline (coefficient = 0.18, p = 0.033) and greater luminal area change (coefficient = 7.0×10^−5^, p = 0.0001) were included in the optimal model for dry macula. Additionally, anti-VEGF (aflibercept, coefficient = 0.056, p = 0.0020), CNV area at baseline (coefficient = 0.034, p = 0.022), and greater luminal area change (coefficient = 6.1×10^−6^, p = 0.033) were included in the optimal model for LogMAR VA change in non-PNV. However, none of these variables were included in the optimal model for dry macula or LogMAR VA change in PNV.

**Table 3:** Baseline and luminal/stromal change parameters included in the optimal model for dry macula or logMAR VA change at 1 month in cases with PNV or non-PNV.

Variables	Dry macula		LogMAR VA change	
	non-PNV	PNV	non-PNV	PNV
	N = 34	N = 21	N = 34	N = 21
Parameters	Coefficient SE P value	Coefficient SE P value	Coefficient SE P value	Coefficient SE P value
**Age (years old)**	NS	NS	NS	NS
**Sex (male)**	NS	NS	NS	NS
**Treatment history**	NS	NS	NS	NS
**Anti-VEGF(increase by aflibercept)**	0.29 0.095 0.005	NS	0.056 0.017 0.0020	NS
**CNV area (mm**^**2**^**)**	0.18 0.08 0.033	NS	0.034 0.014 0 0.022	NS
**Luminal area change (**μ**m** ^**2**^**)**	7.0×10^−5^ 1.5×10^−5^ 0.0001	NS	6.1×10^−6^ 2.7×10^−6^ 0.033	NS
**Stromal area change (**μ**m** ^**2**^**)**	NS	NS	NS	NS
**Luminal area at baseline (**μ**m** ^**2**^**)**	NS	NS	NS	NS
**Stromal area at baseline (**μ**m** ^**2**^**)**	NS	NS	NS	NS

## Discussion

In the present study, we analyzed the short-term changes of choroidal structure after anti-VEGF treatment using EDI OCT, and characterized the distinct response of PNV compared to non-PNV. In patients with non-PNV, we found that greater decrease of luminal area at 1 month, smaller baseline CNV area, and aflibercept compared to ranibizumab treatments were significantly associated with dry macula or better LogMAR VA changes. Considering luminal area decreases after anti-VEGF treatment in non-PNV [20, 21], greater decrease of luminal area could represent greater reduction of CNV activity. We have recently reported that, in patients with non-PNV, upregulation of VEGF in the CNV was less active in eyes with small CNV [22], which would be the reason small CNV is related to higher likelihoods of achieving dry macula and greater LogMAR VA improvement. Aflibercept has a longer intravitreal half-life because of its larger size and a much higher affinity for VEGF-A than ranibizumab, resulting in the greater theoretical duration of biological activity in eyes [23]. Our results were consistent with those of a previous reports describing the effect of aflibercept compared with ranibizumab [24, 25]. In agreement with these previous studies, the current results suggested aflibercept was significantly more associated with dry macula and greater LogMAR VA improvement than ranibizumab, in eyes with non-PNV.

In patients with PNV, however, neither the luminal area at baseline nor decrease in the luminal area was significantly associated with dry macula or LogMAR VA change at 1 month. This might be because, compared to non-PNV, dilation of the luminal area in PNV is not associated with upregulated VEGF induced by active CNV, and the choroidal vessel dilation is not reversed by the inactivation of CNV [12].

The luminal area change after anti-VEGF administration were significantly associated with dry macula rate or greater LogMAR VA improvement in eyes with non-PNV; however, these associations were not observed in PNV. A recent study reported that eyes with active PNV presented with exudative changes in the macula accompanied with visual loss [3]. However, it remains unclear what regulates the activity of PNV. PNV has choroidal vascular abnormalities, such as dilated vessels and hyperpermeability, that could cause exudative changes without CNV as observed in CSC. The activity of CSC was correlated with dilation of the choroidal vessels [26, 27], and it was not VEGF-dependent [28]. These are commonly treated with anti-VEGF agents [5], but had shown distinct outcomes compared to non-PNV. In other words, PNV was associated with an inferior visual outcome after anti-VEGF treatment [6, 8–10]. There was a recent report that suggested that patients with CNV and thickened choroid are refractory to repetitive anti-VEGF administration, but successfully treated with photodynamic therapy [29]. In addition, another recent study showed that VEGF concentrations in the aqueous humor were not increased in PNV and, furthermore, no difference was found in that value between eyes with dry and wet macula in PNV [11, 12] This may suggest the hypothesis that the activity of PNV is modulated, at least in part, by factors other than CNV or its dominant regulator, VEGF, which could lead to the difference in the structural response to anti-VEGF between PNV and non-PNV.

There are several limitations of this study. This was a retrospective non-randomized study, and the numbers of cases were relatively small. A further study would be needed to validate the current results preparing much larger number of samples. The measurements were performed by independent investigators completely masked to the patient information, but they were mostly carried out manually. In addition, the cases had short-term follow-up periods. Additionally, both treatment-naïve patients and the patients with a treatment history were included in the analysis. Although all patients exhibited exudative changes before treatment and multivariate analyses in the present study showed treatment history was not associated with treatment outcomes, possible bias associating with previous treatments could not be fully eliminated. Further analysis including only treatment-naïve patients might be needed to characterize PNV or non-PNV in more detail.

## Conclusions

The change of the luminal area after anti-VEGF administration were significantly associated with dry macula rate or VA improvement in eyes with non-PNV; however, these factors were not chosen as predictors in eyes with PNV. The results of the present study suggested that structural response after anti-VEGF might be different between non-PNV and PNV.

## Supporting information

S1 Dataset The data analysed. (XLSX)
